# Gout Risk Allele Regulating *IRF5* Expression Is Associated with Enhanced IL-1β Production in Response to Palmitate and Monosodium Urate Crystals

**DOI:** 10.3390/ijms26209930

**Published:** 2025-10-12

**Authors:** Valentin Nica, Orsolya Gaal, Medeea Badii, Georgiana Cabău, Andreea-Manuela Mirea, Ioana Hotea, Cristina Pamfil, Simona Rednic, Radu A. Popp, Mihai G. Netea, Tania O. Crișan, Leo A. B. Joosten, HINT Consortium

**Affiliations:** 1 Department of Translational Immunology, Medfuture Institute for Biomedical Research, “Iuliu Hațieganu” University of Medicine and Pharmacy, Str. Pasteur Nr. 6, 400349 Cluj-Napoca, Romania; valetin.nica@umfcluj.ro (V.N.); gaal.ildiko.orsolya@elearn.umfcluj.ro (O.G.); badii.oana.medeea@elearn.umfcluj.ro (M.B.); cabau.georgiana@elearn.umfcluj.ro (G.C.); leo.joosten@radboudumc.nl (L.A.B.J.); 2 Department of Medical Genetics, “Iuliu Hațieganu” University of Medicine and Pharmacy, Str. Pasteur Nr. 6, 400349 Cluj-Napoca, Romaniaanghel.popp@umfcluj.ro (R.A.P.); 3 Department of Internal Medicine, Radboud University Medical Center, Geert Grooteplein Zuid 8, 6525 GA Nijmegen, The Netherlands; mihai.netea@radboudumc.nl; 4 Department of Rheumatology, “Iuliu Hațieganu” University of Medicine and Pharmacy, Str. Clinicilor 2-4, 400006 Cluj-Napoca, Romaniacristinapamfil.umfcluj@elearn.umfcluj.ro (C.P.); srednic@umfcluj.ro (S.R.)

**Keywords:** gout, inflammation, interleukin-1, interferon regulatory factor 5

## Abstract

Interferon Regulatory Factor 5 plays an important role in the regulation of innate immune responses by amplifying the Nuclear Factor κB response, which is critical in gout inflammation. Furthermore, the rs4728141 polymorphism C allele was associated with both increased *IRF5* expression and susceptibility to gout. We examine the association between rs4728141 and cytokine production in response to various Toll-Like Receptor ligands and describe the transcriptomic and proteomic changes observed in patients with gout and controls in relation to this polymorphism. We examine the transcriptome of freshly isolated peripheral blood mononuclear cells (PBMCs) from 93 normouricemic donors and 63 gout patients as well as serum inflammatory proteome in 197 control and 195 gout samples. Stimulation experiments of freshly isolated human PBMCs were performed over 24 h, followed by RNA-sequencing in gout patients and cytokine production measurement by ELISA in normouricemic donors and gout patients. The rs4728141 C allele was associated with increased IL-1β expression in unstimulated PBMCs of controls, but not in gout. No association between the polymorphism and serum inflammatory proteome was found. As expected, an increased *IRF5* expression was observed in stimulated PBMCs of rs4728141 C allele carriers in response to several stimulations. Interestingly, IL-1β production was specifically enhanced in association to the rs4728141 C allele when cells were stimulated with palmitate with or without monosodium urate crystals. This pattern of cytokine production shows a functional impact of rs4728141 in gout through altered IL-1β production.

## 1. Introduction

Gout is a common inflammatory disorder characterized by acute flares of joint pain, swelling, and redness caused by the deposition of monosodium urate (MSU) crystals. While hyperuricemia is a prerequisite for the development of gout, only about a third of individuals with elevated urate will develop gout flares [1]. Furthermore, MSU crystal deposition in joints can also be asymptomatic [2], suggesting that additional triggers are required to start the inflammatory response. Important risk factors for gout and hyperuricemia include sex, age, BMI, and diet, and the conditions are associated with hypertension, chronic kidney disease, diabetes, and heart failure [1]. Genetic factors have been shown to contribute to both hyperuricemia and gout, with most strongly associated polymorphisms located in genes involved in renal urate excretion [3].

Inflammation in gout is primarily an innate immune response mediated by Interleukin-1β (IL-1β). The production and release of IL-1β occur through a two-step process: the first step involves transcriptional upregulation of its precursor, pro-IL-1β, while the second requires cleavage of pro-IL-1β into its active form by caspase-1. Several key pathways are known to regulate the expression of pro-IL-1β and other inflammatory cytokines, most notable of which are Nuclear Factor κB (NF-κB) and mitogen-activated protein kinases, both of which are canonically activated by Pattern Recognition Receptors (PRRs) or IL-1 receptors [4]. The second step required for IL-1β production is the activation of the NLRP3 inflammasome. This multiprotein complex is activated by diverse stimuli—such as extracellular ATP, bacterial toxins, RNA viruses, and other particles—often associated with cellular stress events, such as ionic flux changes, mitochondrial dysfunction, lysosomal damage, and reactive oxygen species (ROS) accumulation [5]. Activation of the NLRP3 inflammasome leads to the cleavage of pro-caspase-1 into active caspase-1, which in turn processes pro-IL-1β into its bioactive form, IL-1β, thereby promoting inflammation.

Interferons (IFNs) are a group of cytokines that play a key role in the immune response to viral infections. However, a growing body of research has also linked the IFN responses to autoimmune diseases, including Systemic Lupus Erythematosus, Dermatomyositis, Rheumatoid Arthritis, Sjögren syndrome, and Systemic Sclerosis [6,7,8]. IFNs exert complex effects on the immune system, with both pro- and anti-inflammatory roles. Type I IFN expression is regulated by a family of nine transcription factors named Interferon Regulatory Factors (IRFs). Among them, *IRF5* is predominantly expressed in bone marrow-derived cells and lymphoid tissues and is recognized for its important proinflammatory role. *IRF5* knockdown was associated with diminished production of inflammatory cytokines TNF and IL-6, macrophage polarization towards anti-inflammatory type M2, and impaired T cell responses. The proinflammatory role of IRF5 was also confirmed in vivo, for example, IRF5—deficient (IFR5^−/−^) mice exhibit significantly reduced joint damage in arthritis models [9,10]. IRF5 is activated by PRRs through the NF-κB pathway as well as by DNA damage. Its activation involves post-translational changes, probably including dimerization and nuclear translocation, followed by binding to target DNA sites [11]. Several mechanisms have been proposed to explain IRF5 function, including synergism with NF-κB at promoters of proinflammatory genes and induction of H3K27 acetylation at multiple enhancer sites. Besides its short-term proinflammatory roles, IRF5 can be involved in regulatory negative feedback mechanisms to control cytokine production. By associating with TRIM28 and histone methyltransferase SETDB1, which can methylate lysine 9 of histone 3 (H3K9me3), the complex can inhibit the expression of TNF [12,13].

A recent genome-wide association study (GWAS) has identified rs4728141 as a lead SNP associated with gout in the European population and included *IRF5* as a high priority gene for involvement in gout [14]. This polymorphism is an intergenic T/C substitution located near the *IRF5* and *KCP* genes. Data from the Genotype-Tissue Expression (GTEx) portal indicate that the alternative allele C is associated with increased *IRF5* expression across multiple tissues, alongside a decrease in *TNPO3* [15]. Given these findings, and the previously documented involvement of IRF5 in proinflammatory events, we hypothesize that the rs4728141 C allele increases the IRF5 expression, which in turn enhances the proinflammatory cytokine production, therefore increasing the probability of gout flares. We aim to further investigate the role of this polymorphism in gout and the general innate immune response by examining the cytokine expression and production patterns in human primary peripheral blood mononuclear cells (PBMCs).

## 2. Results

### 2.1. Presence of rs4728141 Variant C Is Associated with Limited Changes in Transcription of Cytokine Genes in Unstimulated Primary Human PBMCs

**Figure 1:**
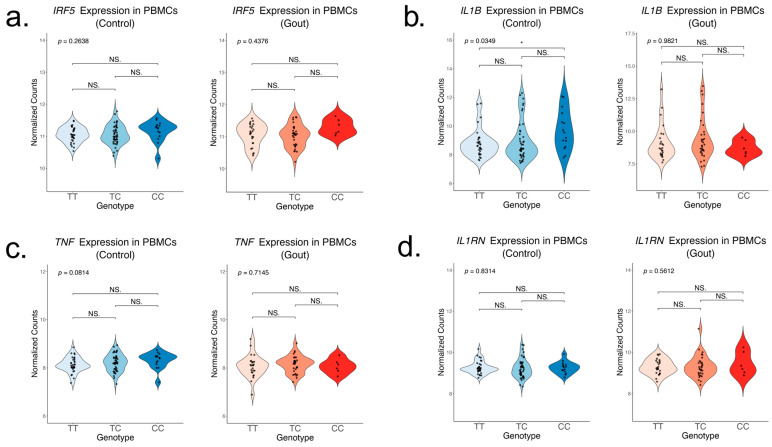
Gene expression of key inflammatory cytokines in unstimulated PBMCs in relation to rs4728141. Expression of *IRF5* (**a**), *IL-1B* (**b**), *TNF* (**c**), and *IL1RN* (**d**) in freshly isolated PBMCs from normouricemic controls (*n* = 93) and gout patients (*n* = 63). The values represent log2 normalized gene counts. The *p* value is obtained after performing a linear regression, and individual groups were compared using the ANOVA and Tukey test. Statistical significance: * *p* <= 0.05; NS. *p* > 0.05.

To assess the association of the rs4728141 SNP with cytokine production, we first examined the mRNA levels of key cytokine genes in freshly isolated human PBMCs in two separate groups that consisted of normouricemic controls (*n* = 93) and patients with gout (*n* = 63). Unstimulated PBMCs depicted no difference in *IRF5* expression in carriers of the gout risk allele C (Figure 1a). Of note, a statistically significant increase in the expression of *IL1B* (Figure 1b) and a limited increase in *TNF* expression (Figure 1c) in the control groups were observed. Meanwhile, the expression of *IL1RN*, encoding the anti-inflammatory cytokine IL-1Ra remained unchanged (Figure 1d).

### 2.2. rs4728141 Is Not Associated with Significant Changes in Serum Cytokines and Chemokines

**Figure 2:**
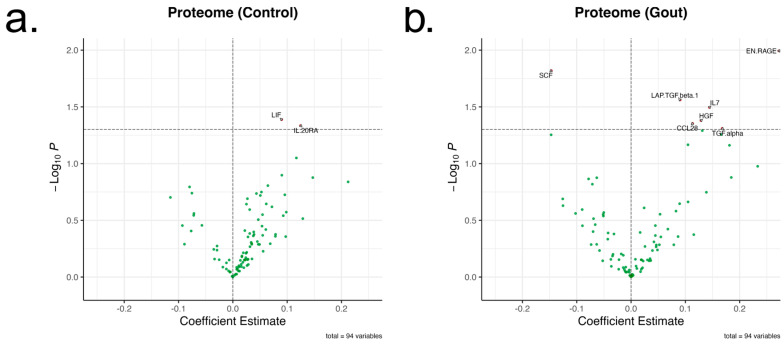
Changes in inflammatory proteome associated with rs4728141. A total of 73 inflammatory proteins in normouricemic control (**a**) and gout (**b**) groups were examined (*n* = 197 and 195). The genotypes were assigned 0, 1, and 2 values, and linear regressions were performed for each protein. The X-axis represents the coefficient estimates and the Y-axis –log_10_ of the nominal *p* values, with the line drawn at *p* = 0.05. No statistically significant changes were observed after multiple testing correction.

Given that rs4728141 was associated with transcriptomic changes in circulating PBMCs, other potential systemic changes associated with the risk allele were investigated. For that purpose, the inflammatory proteome measured in sera of donors using the Olink Inflammation panel [16] was examined. A total of 73 serum proteins were examined in 197 controls and 195 gout patients. The polymorphism was associated with nominally significant changes in several inflammatory proteins, such as S100A12 and IL-7. However, after multiple testing correction, no statistical significance was found (Figure 2a,b).

### 2.3. Transcriptional Analysis in Relation to rs4728141 in Stimulated PBMCs

**Figure 3:**
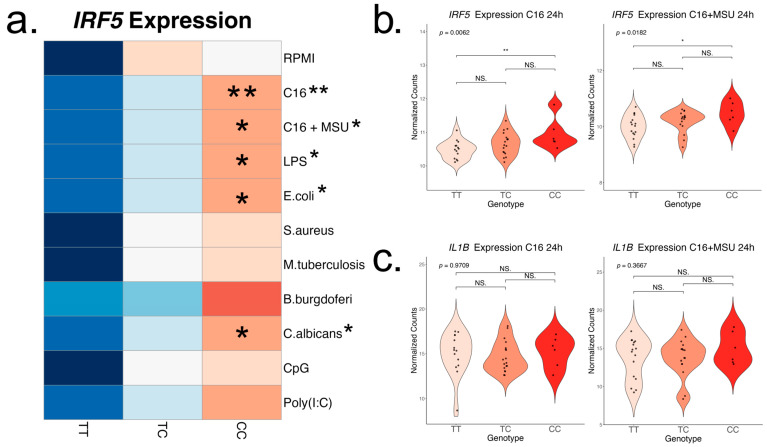
IRF5 and IL-1β expression after in vitro 24 h PBMC stimulation. Cells were stimulated with palmitate (50 μM), with and without the presence of monosodium urate crystals (300 μg/mL), LPS (10 ng/mL), *E. coli* (10^6^/mL), *S. aureus* (10^6^/mL), *M. tuberculosis* lysate (5 μg/mL), *B. burgdorferi* (10^6^/mL), *C. albicans* (10^6^/mL), Poly(I:C) (10 μg/mL), and CpG (1 μg/mL), followed by transcriptomic analysis. (**a**) Row scaled heatmap of IRF5 expression colored by means. Linear regressions were performed to identify if the risk allele is associated with changes in the cytokine production, and the result is indicated next to the stimuli names. In statistically significant associations, heterozygous and homozygous risk allele groups were compared to the homozygous wild-type group (ANOVA and Tukey correction). The strongest correlation was found in the stimulations with palmitate (**b**), and no changes in IL-1β expression were found (**c**). Statistical significance: * *p* <= 0.05; ** *p* <= 0.01; NS. *p* > 0.05.

To assess the changes in the inflammatory response associated with rs4728141, we examined the PBMC response to various PRR stimuli applied in vitro for 24 h. The panel included several PRR agonists: TLR2/4—palmitate (*n* = 38) and palmitate in combination with MSU crystals (*n* = 38), TLR4 agonists—LPS (*n* = 34), *E. coli* (*n* = 41)*,* TLR2 agonists—*S. aureus* (*n* = 40), *M. tuberculosis lysate* (*n* = 41)*, B. burgdorferi* (*n* = 25), Dectin, and other TLRs—*C. albicans* (*n* = 39)*,* TLR9—CpG (*n* = 40) and TLR3—Poly(I:C) (*n* = 40), as well as RPMI control (*n* = 44). Distribution by genotype is reported in the Supplementary Figure S1 and the expression changes in *IL1B* and *IRF5* induced by the stimuli in Supplementary Table S2. We observe a generalized increase in *IRF5* gene expression in rs4728141 C allele carriers, with statistically significant differences in response to stimulations with palmitate, with or without MSU crystals, LPS, *C. albicans*, and *E. coli*, and a similar increase although not statistically significant in all other stimulations (Figure 3a). The strongest association was observed when cells were stimulated with palmitate (Figure 3b). However, no transcriptional differences were observed for *IL1B* (Figure 3c) or other key cytokine genes, such as *IL1RN*, *IL6,* or *TNF* (Supplementary Figure S1).

### 2.4. Rs4728141 Gout Risk Allele C Is Associated with Increased IL-1β Cytokine Release upon Stimulation with Palmitate in Presence or Absence of MSU Crystals

**Figure 4:**
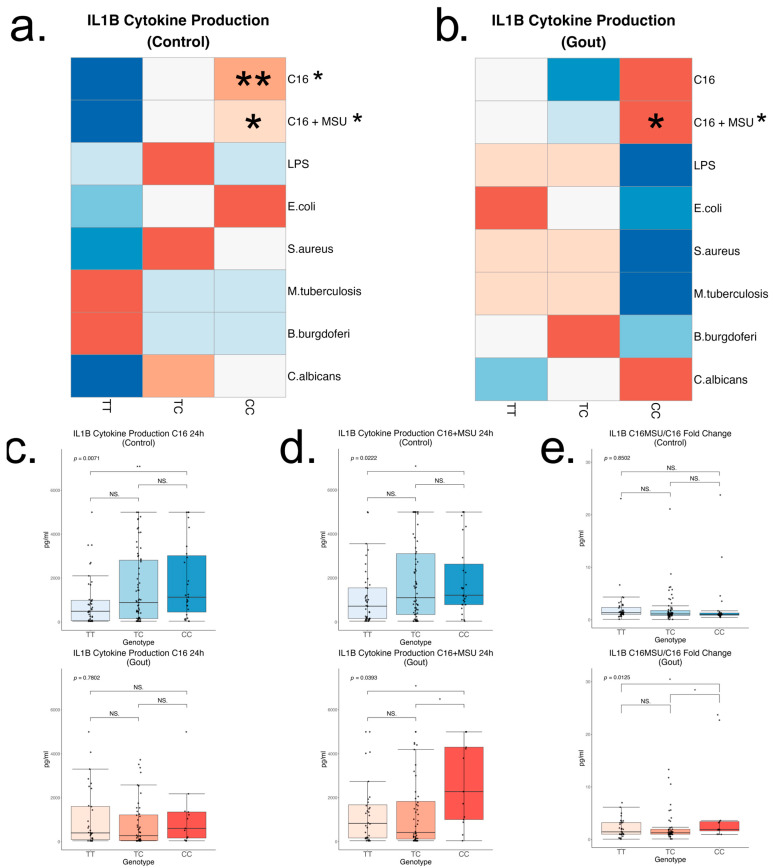
IL-1β cytokine production after in vitro 24 h PBMC stimulation. Cells were stimulated with palmitate (50 μM), with and without the presence of monosodium urate crystals (300 μg/mL), LPS (10 ng/mL), *E. coli* (10^6^/mL), *S. aureus* (10^6^/mL), *M. tuberculosis* lysate (5 μg/mL), *B. burgdorferi* (10^6^/mL), and *C. albicans* (10^6^/mL), followed by cytokine measurements by ELISA in the supernatants. Row scaled heatmaps colored by median. Samples were split into 2 groups—normouricemic controls (**a**) and gout (**b**). Linear regressions were performed to identify if the risk allele is associated with changes in the cytokine production, and the result is indicated next to the stimuli names. In statistically significant associations, heterozygous and homozygous risk allele groups were compared to the homozygous wild-type group (ANOVA and Tukey correction). Boxplots with the key changes represented by palmitate stimulations, with and without MSU, are included separately (**c**,**d**). A ratio of IL-1β production between palmitate, with and without MSU, was calculated for every patient followed by a similar comparison (**e**). Statistical significance: * *p* <= 0.05; ** *p* <= 0.01; NS. *p* > 0.05.

To further assess cytokine production capacity in relation to the rs4728141 SNP, IL-1β, IL-1Ra, and IL-6 concentrations were measured in cell culture supernatants of 24 h stimulated PBMCs in normouricemic controls and gout patients after stimulations with palmitate and palmitate in combination with MSU crystals (*n* = 135 and 93), LPS (*n* = 141 and 101), *E. coli* (*n* = 153 and 110)*, S. aureus* (*n* = 153 and 109), *M. tuberculosis lysate* (*n* = 153 and 110)*, B. burgdorferi* (*n* = 144 and 108), and *C. albicans* (*n* = 153 and 110). Distribution by genotype is reported in the Supplementary Figure S2. Rs4728141 was significantly associated with increased IL-1β production in C allele control carriers, and this was an effect which seemed specific to conditions where palmitate was used, either in the presence or in absence of MSU crystals (Figure 4a–d). The association was weaker in gout patients, where statistical significance was found only in the palmitate in combination with MSU crystals (Figure 4c,d). Furthermore, rs4728141 was associated with an increase in IL-1β production ratio between palmitate with MSU and palmitate alone in gout patients (Figure 4e). The polymorphism was not associated with significant changes in IL-6 or IL-1Ra (Supplementary Figure S2).

## 3. Discussion

Gout pathogenesis involves complex mechanisms that remain largely unresolved, despite significant research efforts. Genetic factors have been linked to gout susceptibility, with the latest GWAS study to date reporting 377 associations with gout [14]. Since hyperuricemia is the key known factor that leads to gout, many of the consistently top-associated genes, such as *SLC2A9*, *SLC22A12*, or *ABCG2,* are involved in urate transport [3]. Given the high prevalence of asymptomatic hyperuricemia, loci specific to gout but not hyperuricemia are of high interest. These include genes that are involved in metabolic processes, such as *FADS1*, *FADS2*, as well as inflammatory responses, such as *IL1RN*, *IL1R1*, *IL6R*, and others. Enrichment of GWAS loci in immune response gene supports the hypothesis that an inflammatory genetic component may contribute to gout beyond hyperuricemia. Major et al. additionally include a prioritization scheme to identify such genes that may be involved in progression from hyperuricemia to gout based on colocalization with publicly available whole blood or monocyte eQTL data, differential expression in gout, association with white blood cell traits, methylation QTL, and previous differential expression studies in monocytes [14]. *IRF5* was one of the top candidate genes, with rs4728141 as the lead SNP at the gout GWAS identified locus in the European population [14]. Besides gout, genetic variants in *IRF5* were already associated with systemic lupus erythematosus [17], rheumatoid arthritis [18], Sjogren syndrome [19], and other inflammatory diseases.

IRF5 is a transcription factor involved in the regulation of inflammatory cytokines production by interacting with key signaling molecules, such as MyD88 and NF-κB subunit p50 [20,21]. This functional role positions IRF5 as a pivotal mediator of innate immune responses. The genetic variant rs4728141, despite being intergenic, is associated with increased *IRF5* expression. Mechanistically, rs4728141 could change the local chromatin accessibility by modifying the affinity to regulatory proteins, as the polymorphism resides within multiple regulatory motifs reported by RegulomeDB [22] or by HaploReg [23]. This DNA region was also shown to be associated with CBX8 in chromatin immunoprecipitation experiments [24]. CBX8 binds to H3K27me3 and is part of the Polycomb Repressive complex 1, a chromatin-based repressor of gene transcription [25,26], further supporting an epigenetic mechanism in the control of *IRF5* expression by rs4728141. Although IRF5 was not thoroughly investigated in the context of gout, its established proinflammatory role in other autoimmune and inflammatory diseases suggests that heightened IRF5 activity could contribute to increased risk and severity of gout. Interferons have been implicated in the response to urate or MSU crystals. A previous study found type I IFN pathway downregulation on transcriptomic assessment of PBMCs exposed to high concentrations of urate, and this signature included a downregulation of *IRF5* as well as its antagonist *IRF4* [27]. In the current study, we observe that a genotype associated with increased *IRF5* expression correlates with elevated IL-1β production following palmitate ± MSU stimulation. Interestingly, despite urate’s inhibitory effect on *IRF5* and IFN signaling, IL-1β levels remained high, suggesting that urate and palmitate may modulate IL-1β through separate pathways, which may not always be mechanistically dependent on IRF5.

Consistent with prior expectations based on the functional role of IRF5, we observed a more potent inflammatory response associated with the rs4728141 risk allele C represented by increased *IL1B* expression in unstimulated PBMCs; however, this effect was observed only in the normouricemic control group. This is likely due to the increased heterogeneity of gout patients, as many variables such as current active gout flares, BMI, current serum urate level, anti-inflammatory medication, and others can significantly alter the systemic immune response. Variability in cell proportions can additionally induce changes in bulk RNA-seq data.

When PBMCs were stimulated with TLR4 ligands for 24 h, we were able to confirm the upregulation of *IRF5* expression associated with the rs4728141 risk allele in most experiments. The trend was consistent across all stimuli, suggesting that increasing the sample size would likely reveal the effect in all experiments. The risk allele was not accompanied by increased expression of cytokines such as *IL1B*, *IL6*, or *TNF*. An important limitation of this study is the lack of data on short-term exposure, which can be one of the main reasons why transcriptomic changes are not observed in these experiments as well as why IL-1β production is only increased in the stimulations with palmitate. The peak expression and production of IL-1β in response to LPS is usually observed at 4 h [28], unlike palmitate that is at 24 h [29,30]. Furthermore, even though we obtained high quality metrics, the imputation process can add some additional errors unlike the conventional genotyping methods.

Interestingly, this study shows the association between the rs4728141 C allele and increased IL-1β cytokine production when PBMCs interacted with palmitate in presence or absence of MSU crystals. This effect seemed to be specific to these stimulations, as no changes in cytokine production were observed in any other stimulation with PRR ligands used in this study. The *IRF5* SNP is associated with significantly enhanced synergistic cytokine production in response to palmitate and MSU crystals, particularly in the gout subgroup, strongly indicating that this variant modulates cytokine production specifically in gout-relevant context.

Palmitate is a saturated fatty acid made of 16 carbon atoms found in most fats and oils. It is also the most common fatty acid found in human blood [31]. Conditions that are typically associated with gout, such as type 2 diabetes, obesity, and dyslipidemia, are also associated with elevated concentrations of fatty acids. Palmitate is currently recognized as a TLR4/TLR2 ligand, which induces an NF-κB-mediated transcriptional response [32]; however, some evidence challenged this assertion [33]. It also activates the NLRP3 inflammasome with the typically associated lysosome destabilization, accumulation of reactive oxygen species, and mitochondrial stress [34]. Palmitate and LPS have also been shown to possess a synergistic proinflammatory effect [35,36]. Unlike LPS, however, palmitate induced upregulation of IL-1β can last over 24 h [30]. Therefore, a potential explanation of our findings is that palmitate stimulations had the best time window to observe the effect of rs4728141 on IL-1β production. Interestingly, there were no SNP-associated changes in *IL1B* mRNA. This discrepancy between unchanged *IL1B* mRNA and elevated IL-1β protein likely reflects additional layers of regulation, such as translational control or secretion dynamics, which are not directly captured by measuring mRNA alone. IRF5 is a transcription factor known to regulate a broad range of proinflammatory cytokines at the transcriptional level; nevertheless, it is plausible that the cytokine responses observed here are controlled by IRF5 through mechanisms beyond mRNA expression changes. IRF5 is involved in metabolic and inflammatory changes specifically in response to palmitate, as its knockdown leads to alterations in tricarboxylic acid cycle and mitochondrial activity [37]. The immune cell metabolic state is strongly linked to cytokine production through epigenetic regulation [38] and activation of the NLRP3 inflammasome [39]. These data suggest a multifaceted role for IRF5 in modulating inflammatory responses.

To conclude, we find that the rs4728141 gout risk allele C is associated with a more potent inflammatory reaction in response to both palmitate and palmitate/MSU crystal combination by upregulating the IL-1β production. Given the central role that IL-1β plays in gout, this increase in cytokine production adds functional evidence to the genetic association of this variant with gout. The rs4728141 C allele may lower the inflammatory threshold, accelerating the shift from silent hyperuricemia to symptomatic gout after MSU crystal formation. This hypothesis requires further investigation in future studies. Therefore, the rs4728141 C allele holds potential as a genetic polymorphism for identifying those at greater risk of developing gout and could play a role in shaping early intervention strategies for gout management.

## 4. Materials and Methods

### 4.1. Participants

The subjects were recruited at the Rheumatology Department of the “Iuliu Haţieganu” University of Medicine and Pharmacy, Cluj-Napoca, Romania, as part of the HINT Project (Hyperuricemia-induced Inflammation: Targeting the Central Role of Uric Acid in Rheumatic and Cardiovascular Diseases, ID P 37 762; MySMIS 103587). Written informed consent was obtained. The study was approved by the Ethical Committee of the “Iuliu Hațieganu” University of Medicine and Pharmacy, Cluj-Napoca (approval no. 425/2016). All study participants in the gout group were included based on the ACR/EULAR 2015 classification criteria, with a minimum score of 8. The control group is represented by individuals from a similar age group, with a serum urate value of less than 7 mg/dl when the cells were obtained. A main set of clinical parameters of participants is presented in Supplementary Table S1.

### 4.2. PBMC Isolation and Stimulation

Peripheral blood was drawn from the cubital vein into EDTA tubes under sterile conditions. PBMCs were separated by Ficoll-Paque density gradient centrifugation and washed 3 times with phosphate-buffer saline solution. The cells were resuspended in culture medium RPMI (Roswell Park Memorial Institute) 1640, supplemented with 50 µg/mL gentamicin, 2 mM L-glutamine, and 1 mM pyruvate. PBMC concentration was adjusted to 10^7^ PBMCs/mL. 10^6^ freshly isolated unstimulated cells were stored in TRIzol for transcriptomic analysis. Stimulation experiments were performed using 0.5 × 10^6^ cells per well, in duplicate, in 96-well plates. Cells were incubated at 37 °C 5% CO_2_ for 24 h with the following stimuli: palmitate (50 μM), palmitate (50 μM) with monosodium urate crystals (300 μg/mL), LPS (10 ng/mL), HK *E. coli* (10^6^ CFU/mL), HK *S. aureus* (10^6^ CFU/mL), *M. tuberculosis* lysate (5 μg/mL), *B. burgdorferi* (10^6^ CFU/mL), heat killed(HK) *C. albicans* (10^6^ CFU/mL), Poly(I:C) (10 μg/mL), CpG (1 μg/mL), and culture medium as negative control. LPS was subjected to ultrapurification before cell culture experiments. Palmitate stock solutions were prepared in ethanol and subsequently conjugated to human albumin (Albuman, approved for intravenous use) prior to cell stimulation. The conjugates were maintained at 37 °C until applying to cells. At the end of each experiment, the supernatants were collected for cytokine measurement, and the remaining cells were stored in 50 μL TRIzol Reagent (Invitrogen) at −80 °C.

### 4.3. Genotyping

Genomic DNA was isolated from whole blood with Wizard^®^ Genomic DNA Purification Kit, Promega (Madison, WI, USA), and genotyping was performed using the Infinium Global Screening Array-24 v1.0 BeadChip, Illumina (San Diego, CA, USA). Initial quality control was performed with Illumina GenomeStudio: The SNPs with <95% call rate were excluded, and all the remaining SNPs were verified and manually re-clustered or removed when clustering was not possible. The data were exported to PLINK format, and further filters were applied: minor allele frequency (MAF) < 0.05; Hardy–Weinberg equilibrium test *p* value < 10^−6^; samples with mismatches between the reported and estimated genetic sex, heterozygosity rate of ±3 standard deviations, and related individuals were excluded. The strands were flipped when required to match GRCh37 hg19. Imputation was then performed on the Michigan Imputation Server with the HRC (Version r1.1 2016) reference. rs4728141 was imputed with an R^2^ of 0.85712.

### 4.4. Transcriptomics

Bulk RNA-sequencing analysis was performed on the DNBseq platform, outsourced to Beijing Genomics Institute, BGI (Copenhagen, Denmark). After assessing the integrity of the RNA with Agilent 2100 Bioanalyzer, the mRNA was enriched with (dT)-attached magnetic beads, followed by reverse transcription to double stranded DNA, 3′ adenylation and adaptor ligation, PCR amplification, denaturation, and cyclization. After initial quality control, which included removal of reads with adaptors, low quality reads (unknown bases > 10% or QC score < 15 in over 50% of the base pairs), and rRNA mapped reads (SOAPnuke v1.5.2), the reads were then mapped to UniGene database (Bowtie2), and the counts were estimated (RSEM v.1.2.12). In freshly isolated PBMC samples, the average number of reads per sample was 32.59 million, with an average clean read ratio of 98.32% and mapping ratio of 94.07%. For the stimulation experiments, the same metrics were at an average of 25.39 million reads, 93.41% clean read ratio, and 77.6% mapping ratio. All samples passed the initial quality control.

The reads were normalized with DESeq2, and additional quality control was performed by removing samples with (1) mismatches between the reported sex and the one estimated by assessing the expression of Y chromosome genes (*DDX3Y*, *USP9Y* and *UTY*), and (2) after performing a Principal Component Analysis, the samples with more than 3 Standard Deviations on principal components 1 and 2. 24 samples were removed as outliers due to an unknown technical error and 8 samples due to sex mismatch. The count data were then exported as normalized counts using the DESeq2 vst function. The PBMC samples were analyzed in 2 batches, and a batch correction was additionally applied using the limma package.

### 4.5. Proteomics

Serum proteomics data from the donors included in this study were retrieved from Cabău et al. [16]. Serum samples were collected at patient inclusion and stored at −80 °C. The stored serum samples were thawed on ice, mixed, and randomized before plating on 96-well PCR microplate, and Olink^®^ Target 96 Inflammation panel was performed. Proteomic assay, data normalization, and quality control were performed at Olink Proteomics (Uppsala, Sweden). Normalized protein expression (NPX) values were examined, and additional quality control filters consisted of over 3 standard deviations from the mean IQR, sample median in distribution plots or deviated in the principal component analysis, samples that were found to be biological duplicates, samples that had missing serum urate data, and proteins that had NPX values below the limit of detection in more than or equal to 20% of samples. The panel included 92 proteins, and after 19 were dropped due to low NPX values, a total of 73 proteins were examined.

### 4.6. Cytokine Measurements

The cytokine concentrations were measured by sandwich ELISA with IL-1β, IL-1Ra, IL-6, and TNF kits, R&D Systems (Minneapolis, MN, USA). Before performing the assay, the samples were diluted 10-fold (IL-1β, IL-1Ra, TNF) or 20-fold (IL-6). Lowest detection range was 78 pg/mL (IL-1β), 390 pg/mL (IL-1Ra), and 94 pg/mL (IL-6).

Samples with detected IL-1β, IL-6, or TNF in RPMI control wells were removed due to suspicion of contamination.

### 4.7. Statistical Analysis

The statistical analysis was performed with R base functions, and figures were generated with ggplot2. When comparing the three genotypes, a linear regression was performed, where the genotypes were given values 0, 1, and 2. Differences between individual groups were assessed by one-way ANOVA and Tukey test, with 0.95 confidence interval.

## Data Availability

Data are accessible upon reasonable request to the corresponding author.

## Supplementary Materials

The following supporting information can be downloaded at: https://www.mdpi.com/article/10.3390/ijms26209930/s1.
