# Supplementary material for: Gout Risk Allele Regulating IRF5 Expression Is Associated with Enhanced IL-1β Production in Response to Palmitate and Monosodium Urate Crystals
Source: Int J Mol Sci. 2025 Oct 12;26(20):9930. doi: 10.3390/ijms26209930 (PMC12563284; doi:10.3390/ijms26209930)
Supplement: Supplementary file 1 [file ijms-26-09930-s001.zip › ijms-3875218-supplementary.pdf]

Supplementary Table 1: Clinical and paraclinical information about the study groups.

	Freshly isolated PBMCs (Transcriptomics)					
Group	Normouricemic controls			Gout		
Genotype	TT	TC	CC	TT	TC	CC
N	32	45	14	26	33	6
Sex (M/F)	13/19	12/33	11/3	20/6	26/7	6/0
Age (Median, Q1-Q3)	63 (54.8-68)	58 (52-68)	59.5 (53-64.5)	63 (58.25-69.75)	60 (51-66)	59.5 (50-75-62.25)
BMI (Median, Q1-Q3)	28.33 (25.57-31.06)	28.29 (26.09-31.51)	26.73 (25.14-28.02)	29.3 (27.35-31.32)	30.79 (27.78-33.95)	29.23 (27.55-30.86)
Type2 Diabetes (%)	9,375	13,33	28,57	23,08	6,06	16,67
Chronic Kidney Disease (%)	0	0	7,14	11,54	18,18	16,67
Serum Urate (Median, Q1-Q3)	5.2 (4.375-5.7)	4.9 (4-5.7)	4.95 (3.65-5.6)	6.3 (5.125-7.475)	6.95 (5.525-9.525)	5.25 (4.725-5.925)
Glucose (Median, Q1-Q3)	97 (89.75-103.75)	95 (86-104)	94 (91-124.5)	105 (100.25-110)	100 (93-107.5)	107.5 (93.75-123.5)
LDL cholesterol (Median, Q1-Q3)	185.5 (172.25-197.5)	135 (104-155)	115.5 (102.25-142.75)	122.5 (97-160)	117 (86-134)	119.5 (112.75-135.25)
CRP (Median, Q1-Q3)	0.185 (0.09-0.525)	0.14 (0.07-0.3)	0.1 (0.09-0.16)	0.4 (0.12-0.6)	0.415 (0.2-0.925)	0.19 (0.11-0.28)
	Stimulation Experiments (Cytokine measurements)					
Group	Normouricemic controls			Gout		
Genotype	TT	TC	CC	TT	TC	CC
N	43	65	27	32	48	13
Sex (M/F)	12/31	44/21	14/13	25/7	39/9	11/2
Age (Median, Q1-Q3)	62 (57-71)	62 (55-69)	59 (55.5-68.5)	61.5 (55-64.5)	62 (54-68)	55 (50-64)
BMI (Median, Q1-Q3)	28.69 (24.65-31.99)	28.29 (26.45-31.54)	27.55 (26.27-30.75)	29.07 (26.6-32.33)	30.1 (26.78-32.96)	31.22 (27.17-33.65)
Type2 Diabetes (%)	9,3	15,38	14,81	9,375	18,75	15,38

Chronic Kidney Disease (%)	0	0	0	6,25	10,42	7,69
Serum Urate (Median, Q1-Q3)	5.2 (4.25-5.75)	5.2 (4.3-5.9)	4.85 (4.3-5.475)	6.65 (5.35-7.95)	6.7 (5.75-8.15)	7.75 (6.225-8.773)
Glucose (Median, Q1-Q3)	93 (89-106.5)	100 (87-112)	94.5 (90-112.25)	102 (94.75-110)	103 (94-115.5)	98 (94-119)
LDL cholesterol (Median, Q1-Q3)	132 (117.5-157)	125 (98-152)	129 (106-143)	118.5 (99.5-132.25)	107 (88.75-144.5)	118 (113-140)
CRP (Median, Q1-Q3)	0.17 (0.1-0.5)	0.2 (0.1-0.4)	0.12 (0.1-0.3)	0.3 (0.185-0.5)	0.345 (0.12-0.775)	0.5 (0.2-0.6)

Supplementary Table 2: IL1B and IRF5 expression changes in response to PRR ligands.

Stimuli (compared to RPMI)	Gene	baseMean	log2FoldChange	pvalue	padj
Palmitate	IRF5	1639,788158	0,127386227	0,069976846	0,110739437
LPS	IRF5	1484,071359	-0,304064913	5,78346E-06	1,18239E-05
C.albicans	IRF5	2083,224327	0,605204806	2,27799E-17	9,01309E-17
S.aureus	IRF5	1959,69632	0,520218043	1,76462E-13	5,36192E-13
E.coli	IRF5	1721,099294	0,210416645	0,000870704	0,001427146
B.burdoferi	IRF5	1605,116699	0,092865515	0,320809321	0,371893437
M.tuberculosis	IRF5	1726,148485	0,215217719	0,004945391	0,006726746
Poly(I:C)	IRF5	3179,990866	1,678102973	4,923E-121	1,0116E-119
CpG	IRF5	3069,982541	1,491666629	3,81266E-79	1,66671E-77
Palmitate	IL1B	32236,40453	5,316200837	2,26151E-73	8,71058E-71
LPS	IL1B	126830,4523	8,022070843	0	0
C.albicans	IL1B	63459,01629	7,023193556	3,0081E-305	1,3893E-301

S.aureus	IL1B	83754,65851	7,40016657	0	0
E.coli	IL1B	81560,82354	7,139362757	1,3775E-248	9,5462E-245
B.burdoferi	IL1B	113170,6906	7,429714445	3,7279E-189	4,7049E-186
M.tuberculosis	IL1B	163963,2441	8,481821757	6,0778E-147	3,4952E-145
Poly(I:C)	IL1B	2551,989162	1,588567944	1,59486E-12	3,09324E-12
CpG	IL1B	2377,073005	-0,496504307	0,02615354	0,035035396

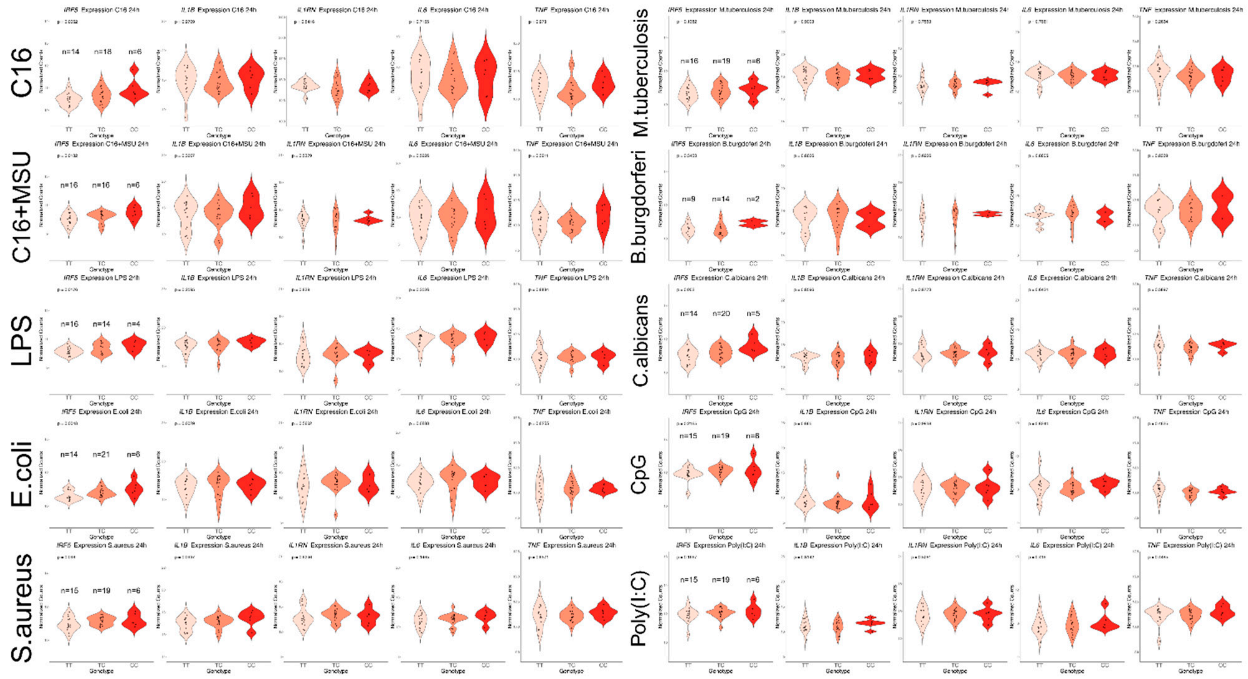

Supplementary Figure S1: rs4728141 effect on key cytokine expression. The PBMC response to palmitate, palmitate+MSU, LPS, *E.coli*, *S.aureus*, *M.tuberculosis*, *B.burgdorferi*, *C.albicans*, CpG, and Poly(I:C) was evaluated after 24 hours by RNA-seq. The normalized expression values of *IRF5*, *IL1B*, *IL1RN*, *IL6*, and *TNF* are reported. The sample size for each experiment is shown in the first figure (IRF5). The p values for linear regressions where the genotypes are assigned values 0, 1, and 2 are reported.

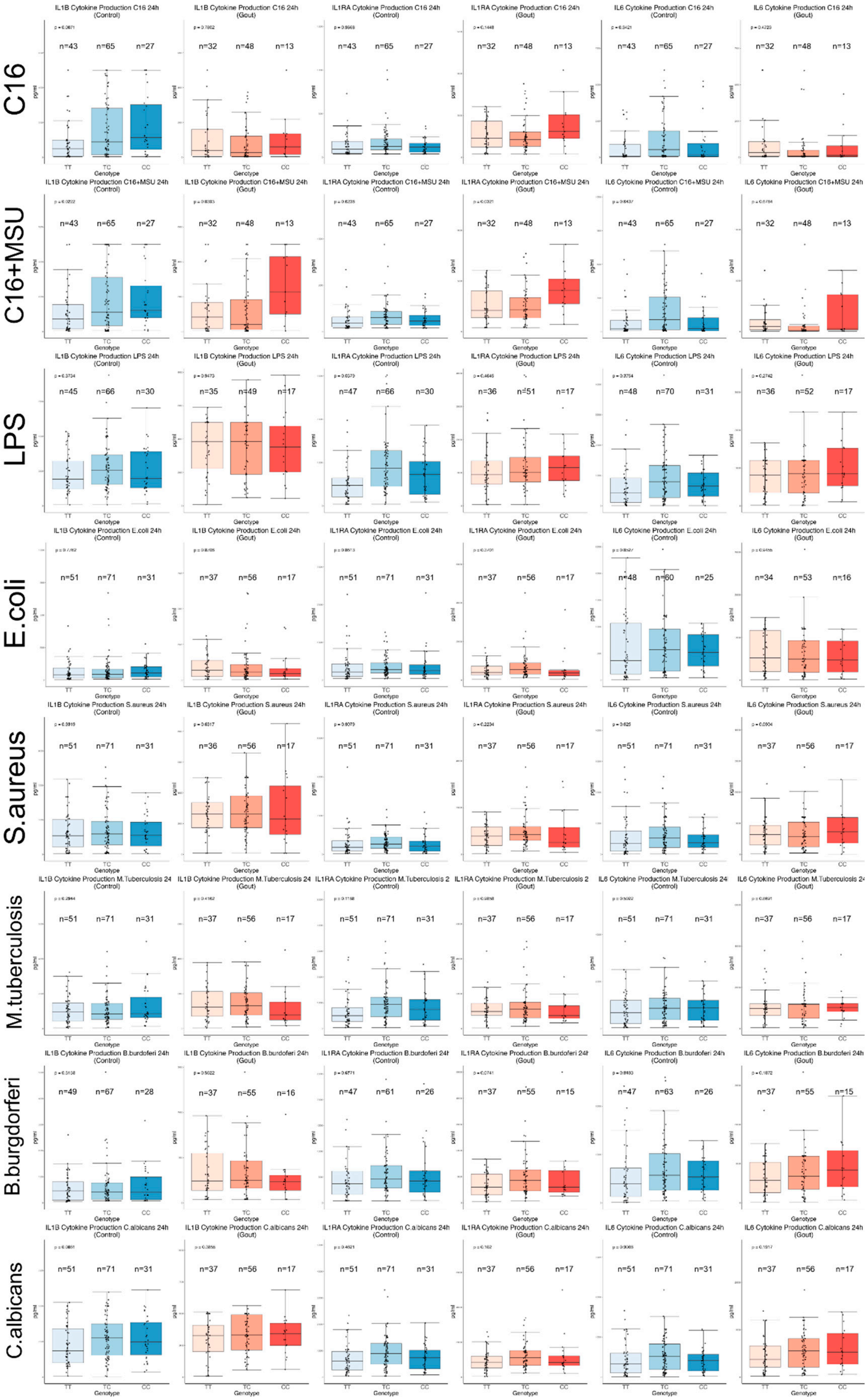
C16
C16+MSU
LPS
E.coli
S.aureus
M.tuberculosis
B.burgdorferi
C.albicans
IL1B Cytokine Production C16 24h (Control)
IL1B Cytokine Production C16 24h (Gout)
IL1RA Cytokine Production C16 24h (Control)
IL1RA Cytokine Production C16 24h (Gout)
IL6 Cytokine Production C16 24h (Control)
IL6 Cytokine Production C16 24h (Gout)
IL1B Cytokine Production C16+MSU 24h (Control)
IL1B Cytokine Production C16+MSU 24h (Gout)
IL1RA Cytokine Production C16+MSU 24h (Control)
IL1RA Cytokine Production C16+MSU 24h (Gout)
IL6 Cytokine Production C16+MSU 24h (Control)
IL6 Cytokine Production C16+MSU 24h (Gout)
IL1B Cytokine Production LPS 24h (Control)
IL1B Cytokine Production LPS 24h (Gout)
IL1RA Cytokine Production LPS 24h (Control)
IL1RA Cytokine Production LPS 24h (Gout)
IL6 Cytokine Production LPS 24h (Control)
IL6 Cytokine Production LPS 24h (Gout)
IL1B Cytokine Production E.coli 24h (Control)
IL1B Cytokine Production E.coli 24h (Gout)
IL1RA Cytokine Production E.coli 24h (Control)
IL1RA Cytokine Production E.coli 24h (Gout)
IL6 Cytokine Production E.coli 24h (Control)
IL6 Cytokine Production E.coli 24h (Gout)
IL1B Cytokine Production S.aureus 24h (Control)
IL1B Cytokine Production S.aureus 24h (Gout)
IL1RA Cytokine Production S.aureus 24h (Control)
IL1RA Cytokine Production S.aureus 24h (Gout)
IL6 Cytokine Production S.aureus 24h (Control)
IL6 Cytokine Production S.aureus 24h (Gout)
IL1B Cytokine Production M.Tuberculosis 24 (Control)
IL1B Cytokine Production M.Tuberculosis 24 (Gout)
IL1RA Cytokine Production M.Tuberculosis 2 (Control)
IL1RA Cytokine Production M.Tuberculosis 2 (Gout)
IL6 Cytokine Production M.Tuberculosis 24 (Control)
IL6 Cytokine Production M.Tuberculosis 2 (Gout)
IL1B Cytokine Production B.burdoferi 24h (Control)
IL1B Cytokine Production B.burdoferi 24h (Gout)
IL1RA Cytokine Production B.burdoferi 24h (Control)
IL1RA Cytokine Production B.burdoferi 24h (Gout)
IL6 Cytokine Production B.burdoferi 24h (Control)
IL6 Cytokine Production B.burdoferi 24h (Gout)
IL1B Cytokine Production C.albicans 24h (Control)
IL1B Cytokine Production C.albicans 24h (Gout)
IL1RA Cytokine Production C.albicans 24h (Control)
IL1RA Cytokine Production C.albicans 24h (Gout)
IL6 Cytokine Production C.albicans 24h (Control)
IL6 Cytokine Production C.albicans 24h (Gout)
TT
TC
CC
Genotype
pg/ml

Supplementary Figure S2: rs4728141 effect on cytokine production. The PBMC response to palmitate, palmitate+MSU, LPS, *E.coli*, *S.aureus*, *M.tuberculosis*, *B.burgdorferi*, and *C.albicans* was evaluated after 24 hours. IL1B, IL1RA, and IL6 concentrations were measured in supernatants by ELISA (pg/ml). The sample size for each experiment is shown in each panel. The p values for linear regressions where the genotypes are assigned values 0, 1, and 2 are reported.
